# An Efficient and Lightweight Illumination Model for Planetary Bodies Including Direct and Diffuse Radiation

**DOI:** 10.3390/jimaging6090084

**Published:** 2020-08-24

**Authors:** Marco Scharringhausen, Lars Witte

**Affiliations:** Institute of Space Systems, German Aerospace Center, 28359 Bremen, Germany; Lars.Witte@dlr.de

**Keywords:** surface properties, planetary bodies, illumination model, ray tracing, multispectral, inversion

## Abstract

We present a numerical illumination model to calculate direct as well as diffuse or Hapke scattered radiation scenarios on arbitrary planetary surfaces. This includes small body surfaces such as main belt asteroids as well as e.g., the lunar surface. The model is based on the ray tracing method. This method is not restricted to spherical or ellipsoidal shapes but digital terrain data of arbitrary spatial resolution can be fed into the model. Solar radiation is the source of direct radiation, wavelength-dependent effects (e.g. albedo) can be accounted for. Mutual illumination of individual bodies in implemented (e.g. in binary or multiple systems) as well as self-illumination (e.g. crater floors by crater walls) by diffuse or Hapke radiation. The model is validated by statistical methods. A $\chi^{2}$ test is utilized to compare simulated images with DAWN images acquired during the survey phase at small body 4 Vesta and to successfully prove its validity.

## 1. Introduction

Reliable prediction or at least estimation of illumination conditions on the surface of planetary bides (e.g., the Earth moon) or small bodies like asteroids or comet cores is essential for mission planning purposes.

In general, mission planning for any vehicle on a strategic (before start of the mission) and/or tactical level (during the mission) requires knowledge about environmental conditions at the destination. From a general point of view and depending on the vehicle(s) involved, this might include atmospheric properties such as density, wind speed, as well as surface properties such as roughness, slope, gravity, and illumination. This applies not only to surface elements but also to planetary landers.

Firstly, thermal design of any lander space craft relies on a reliable information about the environment (e.g., [1]). As solar radiation is in general the major source of energy on the surface of a small bodies without atmosphere in space, a simulation suite for calculation of the surface radiative intensity delivers boundary conditions for any thermal model of the surface.

**Figure 1:**
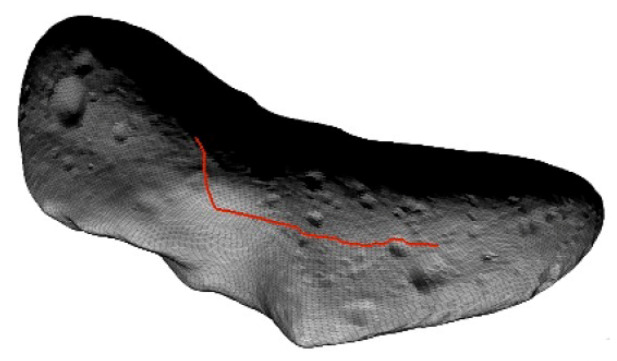
Optimal path of some generic rover on the surface of (433) Eros. The cost function involves illumination, surface roughness, and slope to equal weights. The light source (point) is located at the bottom left hand side corner, shading indicates illumination (see [4] for shape model).

The same applies to the design of a mobile surface element powered by solar cells, e.g., a rover. For rovers, additional aspects come into play, i.e., not only the thermal and power budget are affected by illumination conditions but also the path and trajectory planning of the rover itself ([2,3]). Being able to reliably predict illumination at any time and at any point on the surface of the small or planetary body is an essential part of the cost function in any optimization process. During the mission, the rover’s mission plan may involve travel to several distinct sites, interleaving periods of dedicated science data collection with periods of traversal and opportunistic science. To repower internal batteries, it might be beneficial to pause for a period of time to exploit time slots of maximal illumination. To plan all this in advance at least on a tactical, if not on a strategic level, an illumination simulation is crucial. In addition, it may be beneficial to have the illumination simulation as lightweight as possible to have it run onboard the rover (see e.g. Figure 1).

In addition, fundamental research questions rely on high-quality estimates of the radiation and thermal environment on the body surface. This applies, e.g., to the formation of cometary tails and halos but also to the varying ice content of asteroids.

## 2. Illumination Analysis: State of the Art and Motivation

To date, a number of illumination simulation tools and suites are accessible for the scientific community. Among those are elaborated tools as the PANGU (Planet and Asteroid Natural Scene Generation Utility) tool [5,6] for simulation and visualization of planetary surfaces. PANGU’s development goal is to support the development of planetary landers (with optical navigation) to navigate towards the surface and to perform hazard avoidance near the landing site. PANGU can be used to generate an artificial surface representative of cratered planets and to provide images of the simulated planet. Using position and attitude of a spacecraft or its sensors, respectively, PANGU generates an image of the surface as seen from that camera position. From its beginnings, PANGU’s capabilities have been extended from the lunar surface towards the martian surface as well as space scenario imaging, it has been applied to e.g., New Horizons or Mars VMC. Recent publications (2014–2018) for PANGU comprise [7,8,9,10]. These include a description of functionalities such as inclusion of different bidirectional reflectance functions (Hapke, Oren-Nayar, and Lunar Lambert). In addition, performance has been improved to support real-time image generation. A fast and powerful GPU-based camera model which includes geometric distortions and sensor/radiometric noises was introduced in PANGU v3.60 (2016).

Another illumination model is presented in [11]. This publication presents a simulation model for illumination conditions at the lunar south pole, based on LOLA DEM data. This tool presents a forward model focused on in-situ illumination conditions for purposes of mission planning and landing site selection. It does not generate camera or radiance images.

Similarly, Ref. [12] presents an algorithm whose purpose is to generate local illumination conditions on an underlying shape model. In contrast to [11], however, a uncertainty analysis is possible. That is, map tie errors’ impact on local irradiances can be estimated. That is, uncertainties in the shape model or the elevation map such as mis-estimates of the extension/height/depth of surface features can be transferred to estimates of uncertainties in the local irradiance. Ref. [13] describes an approach to incorporate inverse modeling similar to our method. The forward model to generate artificial camera images is embedded in a “wrapping“ optimization algorithm to minimize deviations of the simulated from actually acquired camera images. Surface properties such as brightness are independent variables, thus providing an estimate of those after convergence of the optimization to some local or global minimum.

Another (proprietary) tool named SurRender [14] is available on the market. This is a software toolkit for the scientific-level generation of space scene images. It is proprietary software of Airbus Defence and Space, and its primary data product are simulated camera images. Physically-accurate images are produced with quantitative radiometric values, expressed in physical units, i.e., irradiance value per pixel. In addition, SurRender is able to include textures on a planetary scale. These textures can be user-provided by external data as well as internally calculated by SurRender. The latter case then yields procedural textures. Textures can be mapped to digital terrain models.

The image generation method is ray tracing, as is the case for our tool. Diffraction by atmospheres as well as multiple scattered light (both by atmosphere and ground) can be included in the images. In addition, arbitrary bidirectional reflectance functions (BDRF) such as Lambert, Hapke (for regolith-covered moons and asteroids), Phong, and Oren-Nayar (for Jovian moons) can be chosen by the user. Additionally, a sensor model can be delivered by the user, including geometry, optics, motion, sensor, noise, and electronics.

The development goal of the illumination model presented in this study is not to design a tool superior to PANGU, SurRender, or other tools, since these tools have a long history and a large team of highly-skilled developers. The aim of the illumination tool presented here is to be as lightweight as possible from a code base point of view, being open-source and easily being accessible for programmers and developers (e.g., students). It shall be designed to act as an educational tool for students of physics, information engineering, or mathematics to learn about illumination simulation theory.

## 3. Illumination Analysis: The SLIM Model

The Space-Scene Lightweight Illumination Model (SLIM) is designed to be lightweight and efficiently deliver illumination intensities on the surface ot a planetary body. Its primary data product is thus not a camera image, but the physical radiance or irradiance on the surface of any body. The camera images generated for this study are used for validation against real remote sensing data (e.g., DAWN mission). Point measurements of illumination have been recorded from various lander missions; however, this has never been done on a near-global scale.

SLIM offers the option to do inversion, i.e., extraction of optical surface properties-Hapke parameters or albedo-from camera images. This inversion can be done not only for disk-integrated data sets but surface parameters can be calculated but also on scales of a couple of triangles.

Illumination calculated by SLIM can be fed directly into a thermal model or any vehicle power model, since the data are exported in general ASCII format. The SLIM model is versatile since surfaces can be handed to the model as triangulations given in PLY, STL, or OBJ format.

**Figure 2:**
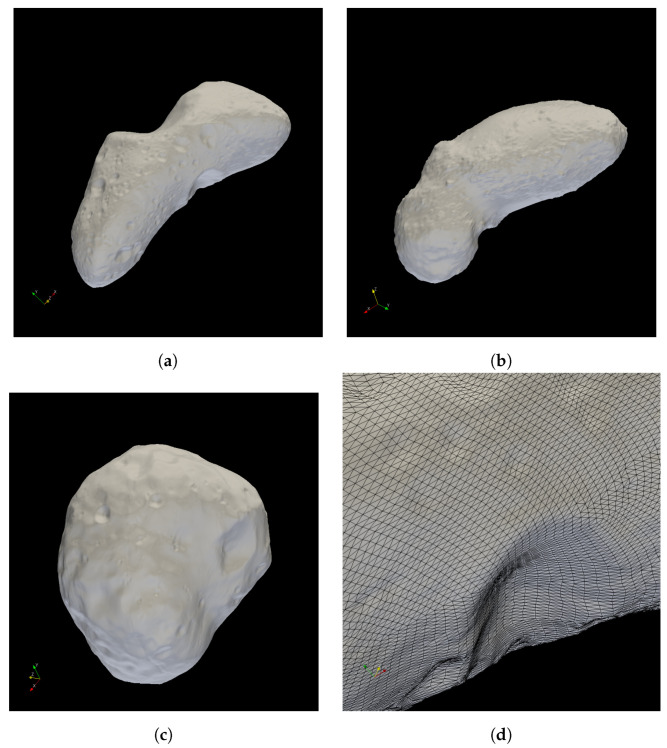
Surface triangulations of small bodies Eros, Itokawa and Phobos. Average edge lengths are calculated from the average triangle area, assuming equilateral triangles. Thus, the values for average edge lengths are of a correct order of magnitude. (**a**) Asteroid (433) Eros. 196,608 faces, average triangle area 5771 m^2^, average edge length approx. 115 m [16]; (**b**) Asteroid (25143) Itokawa, 196,608 faces, average triangle area 2 m^2^, average edge length approx. 2 m [4]; (**c**) Martian satellite Phobos, 49,152 faces, average triangle area 33,500 m^2^, average edge length approx. 278 m [17]; (**d**) close-up of triangle patches on Eros’s surface, average triangle area 22,982 m^2^, average edge length approx. 230 m [17].

Surfaces do not need to be closed and can thus be small patches of any body surface. Bodies of arbitrary size (asteroids, moons, planets) can be handled with the same code and user interface. Crater and boulder distributions cannot be parametrized but need to be explicitly provided (as part of the triangulated surface) by the user. It should be noted, however, that the planetary surface itself and the boulders/rocks on top of it can be input in separate files. There is no need to merge the two triangulations beforehand. Surfaces can be of arbitrary shape, i.e., do not need to be convex but can have edges, bulges, juts, and overhangs; consider, e.g., (25,143) Itokawa (Figure 2b).

Surfaces can have arbitrary spatial resolution, and triangles of almost (up to numerical limits) sizes can be handled simultaneously in the same data set.

The main benefit in terms of the forward (raytracing) feature of SLIM compared to other ray tracing methods is the small code footprint (both source code as well as during runtime) as well as it runtime speed. In addition, the code is easily accessible and can be utilized for educational purposes.

## 4. Small Body Data Model

The small body data used in this study come as point coordinates of surface points in $R^{3}$ along with a triangulation. No points or triangles are located in the interior of the bodies. The number of triangles patches can vary from a few hundred up to a few million, depending on data availability and requirements on the accuracy of the illumination analysis.

The image resolution is constant at 1024 × 1024 pixels. The Vesta shape model (compare Section 4) is available in four different resolutions (see Table 1).

Data can be gained from remote sensing methods such as earth-based radar observations (as is the case e.g., for Phobos) as well as from close-encounter orbiter data (LIDAR, etc.), as is the case, e.g., for (25,143) Itokawa, see Figure 2.

## 5. Ephemerides

All relevant ephemerides data of body, spacecraft and sun are calculated by the SPICE toolkit vN0066 [18]. This yields the position of the S/C as well as the line-to-the-sun (LTS) and the line-of-sight (LOS) of the camera in the body-fixed coordinate system.

For the case study presented later, imagery data 4 Vesta as acquired by the Dawn mission have been utilized and ephemerides data are given in the Dawn-Claudia coordinate system for Vesta. The kernel files used in this study are listed below (see Table 2).

## 6. Radiation

The sun is considered the only source of direct radiation. Bodies are assumed to be able to illuminate each other (e.g., in binary systems) or themselves (e.g., crater floors by crater walls) by diffuse radiation. Lambertian diffuse scattering can be accounted for up to arbitrary orders of scattering by using the method of radiosity ([19,20,21,22]). For diffuse scattering following a non-Lambertian reflectance function, only the first order of scattering is accounted for.

**Figure 3:**
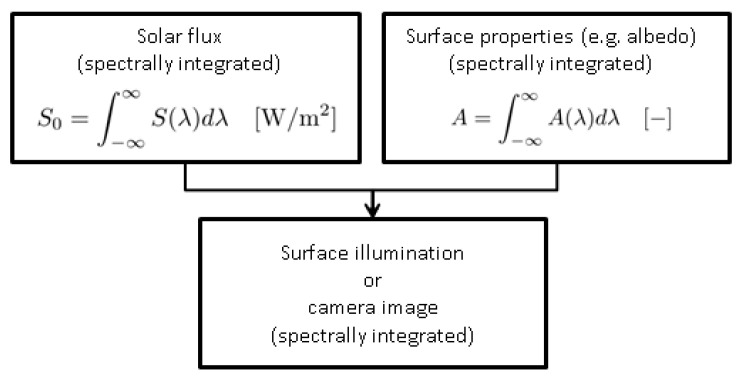
Generation of spectrally integrated images. All relevant parameters such as solar flux or optical properties of the surface are averaged and then used in the algorithm.

**Figure 4:**
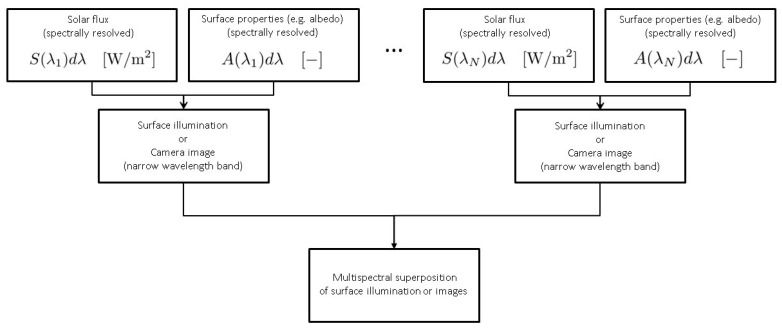
Multispectral superposition of surface illumination or camera images. Surface illumination levels or camera images are calculated for *N* wavelengths, respectively, wavelength bands (or arbitrary width) and then added. This approach is numerically more expensive but yields more accurate results.

All quantities are considered to be independent of wavelength. This is an approximation to reality, e.g., albedo (see Section 6.2) is wavelength dependent. For practical purposes, making quantities wavelength independent can be accomplished by averaging over a certain wavelength range. Alternatively, the illumination algorithm presented here can be used for single wavelengths or wavelength intervals and the results super-positioned afterwards. That is, "color" images can be rendered by individually rendering monochromatic images using different values for relevant physical quantities such as albedo, Hapke parameters, etc. The corresponding set of monochromatic images can then be super-positioned to yield a polychromatic image. For the calculation of surface illumination, intensities of radiation can be calculated for a predefined set of wavelengths and then added to yield the multispectral illumination, see Figure 3 and Figure 4.

### 6.1. Direct Radiation

**Figure 5:**
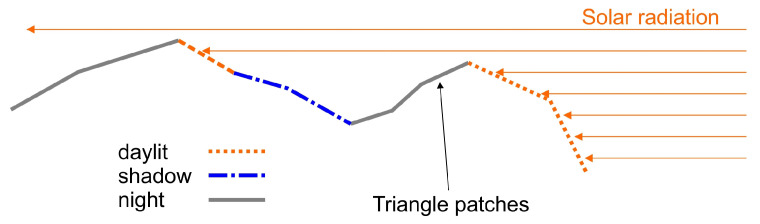
Direct radiation on the body surface. There are three types of triangles: day, night, and shadow.

Direct solar radiation is calculated using ray tracing on a body-fixed Cartesian grid. At the beginning of all calculations, a Cartesian grid with a user-defined number of boxes in the *x*-, *y*-, *z*-directions is generated. The boundaries of this grid coincide with the axis-aligned bounding box (AABB) of the body under consideration. The *home box* of a triangle is referred to as the box that the center of gravity of this triangle is located in. To determine which triangle patches are in daylight or in shadow (see Figure 5), the solar ray is traced through the grid (see Figure 5). The ray is not tested against intersection with all surface patches but only with those in the respective grid cell (starting in the triangle’s home box). If there is no intersection in an individual grid cell, the ray proceeds to the next and the triangle patches in that cell are tested.

During the algorithm, the following pseudocode is executed:

 

# Triangle Irradiances are denoted by R(1..N) (N = number of triangles)

 

for i=1 \dots N (number of triangles) do

 

p    = cog of triangle #i

n(i) = outer normal vector if tri #i

h(i) = home box of triangle

# Ray towards the sun

g(t) = p + t * s/s

 

# Calculate dot product between outer normal and ray vector

# to determine "night" triangles

 

if s * n(i) <= 0 then

# sun is below local horizon

R(i) = 0

cycle to next triangle

end~if

 

# Trace the ray through the grid

# Triangle #i is sunlit if ray g leaves the~AABB

 

current box =  h(i)

until ray left AABB~do

 

check all triangles <> #i in current box for intersection with ray~g

 

if intersect = 1 then

# Triangle #i is shadowed by another triangle

R(i) = 0

cycle to next triangle

else if intersect = 0 then

# Triangle #i is not shadowed by any triangle in the current box

determine wether ray left current box in +x, -x, +y, -y, +z, -z direction

proceed to next box

end~if

 

end~until

 

# Calculate irradiance as dot product between sun vector and

# outer triangle normal

R(i) = s * n(i)

 

end~do

 

At the end of the algorithm, the following irradiance values R(i),i=1,⋯,N have been calculated from the solar flux vector $\vec{s}$ and the triangles’ outer normal vectors $\vec{n}\left(i\right)$:

(1) $$\begin{array}{ccc} \mu & = & \langle \vec{s},\vec{n}\left(i\right)\rangle \end{array}$$

(2) $$\begin{array}{ccc} R(i) & = & \left\{\begin{array}{cc} \mu & ,\;\mu \geq 0,i.e.,\;\mathrm{triangle}\;\mathrm{is}\;\mathrm{in}\;\mathrm{daylight},\;\mathrm{sun}\;\mathrm{above}\;\mathrm{local}\;\mathrm{horizon}\\ 0 & ,\;\mu <0,i.e.,\;\mathrm{sun}\;\mathrm{below}\;\mathrm{local}\;\mathrm{horizon}\\ 0 & ,\;\mathrm{triangle}\;\mathrm{is}\;\mathrm{shadowed}\;\mathrm{by}\;\mathrm{another}\;\mathrm{triangle} \end{array}\right. \end{array}$$

### 6.2. Backscatter and Diffuse Radiation

Backscattered light (onto the camera) as well as diffuse radiation (illuminating areas on the body surface that are shadowed in direct light) can be calculated as totally diffuse, i.e., Lambertian reflectance. Alternatively, scattering according to the Hapke reflectance model can be applied ([23]).

The Hapke bidirectional reflectance model has been widely used for modeling of atmosphereless planetary soil surfaces covered with regolith. This covers e.g., the lunar surface and the surface of Mercury as well as surfaces of small bodies like Phobos, Vesta, Ceres, etc. Most of those surfaces are characterized by low single-scattering albedos and low degrees of anisotropies. However, effects such as opposition surge might occur that is a strong increase in backscattered irradiance for phase angles of approximately zero. This effect can be accounted for in the Hapke model by a "hot spot" correction, parametrized by (angular) width and peak height. Hapke built his model on approximate H-functions developed by Chandrasekhar [23] and derives from this a simple parametrization of the radiation that is multiply scattered in the sub-surface soil region. From this, a bidirectional reflectance function is derived that describes apparent reflectance of the soil surface.

Diffuse scattering can be accounted for up to arbitrary orders of scattering by using the method of radiosity ([19,20,21,22]). Radiosity is based on the concept of conservation of energy, i.e., incident energy flux on a triangle equals outgoing flux. Radiosity quantifies this idea.

The scattered radiative power dE into solid angle dω from a surface patch dA as seen from angle ϕ against the surface normal is given by:

(3) $$\begin{array}{ccc} dE & = & I\cos(\phi )d\omega \end{array}$$

Here, *I* is the constant intensity of radiation in all directions. Given diffuse scattering, the radiated power *P* (in W/m${}^{2}$) of a surface patch dA (here: a triangle patch), consists of two contributions, the self-emitted power *E* as well as the reflected power ρR ([19]):

(4) $$\begin{array}{ccc} P_{i} & = & E_{i}+\rho_{i}R_{i}\;,\;i=1,\cdots ,N \end{array}$$

Here, ρ∈[0,1] is the reflectivity (albedo) of the surface, being 0 for a totally black surface and 1 for perfect diffuse reflection. The incident flux $R_{i}$ is the sum of all radiated powers of all other triangles weighted by form factors $F_{ij}$ that quantify the mutual visibility and viewing geometry:

(5) $$\begin{array}{ccc} R_{i} & = & \sum_{j=1,j\neq i}^{N}P_{i}F_{ij}\;,\;1,\cdots ,N \end{array}$$

(6) $$\begin{array}{ccc} P_{i} & = & E_{i}+\rho_{i}\sum_{j=1,j\neq i}^{N}P_{i}F_{ij}\;,\;1,\cdots ,N \end{array}$$

**Figure 6:**
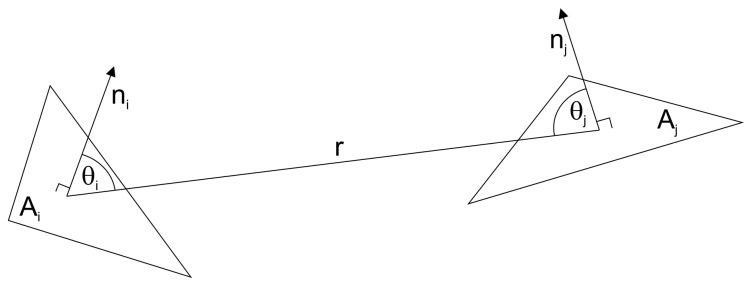
Calculation of form factors $F_{ij}$ (own work).

Given triangles #i and #j, let $\theta_{i}$ and $\theta_{j}$ be the angles between the respective outer normal vectors ${\vec{n}}_{i},{\vec{n}}_{j}$ and the line connecting the centers of gravity of the two triangles. Let *r* be the distance of the cog’s. We consider the triangles small w.r.t. to the surface of the small body (typically, the surface is patched with a couple of thousand triangles) as well as plane (this is trivial). Thus, integration over the triangle surfaces is not necessary and the following simplified form of the form factor calculation can be used (see Figure 6):

(7) $$\begin{array}{c} F_{ij}=\left\{\begin{array}{cc} \frac{\cos\left(\theta_{i}\right)\cos\left(\theta_{j}\right)A_{j}}{\pi r^{2}} & \;,\;\mathrm{tri}\#j\mathrm{is}\mathrm{visible}\mathrm{from}\mathrm{tri}\#i\\ 0 & \;,\;\mathrm{tri}\#j\mathrm{is}\mathrm{not}\mathrm{visible}\mathrm{from}\mathrm{tri}\#i \end{array}\right. \end{array}$$

Note that

(8) $$\begin{array}{ccc} A_{i}F_{ij} & = & A_{j}F_{ji} \end{array}$$

Equation (6) constitutes a system of linear equations in *N* unknowns, the total outbound radiative powers $P_{i}$ of the individual triangle patches. Since *N* can be large, it is numerically unfavorable to handle the $N^{2}$ coefficient matrix and solve the system directly. It is sparse, so that iterative solvers are favorable. One of those is the Jacobi method. It can be used in parallel computing mode and has the convenient feature that the iterations correspond to the individual orders of scattering, i.e., after the *k*-th iteration, scattering of order up to *k* is accounted for. Usually, iterations are ended after a predefined number $K_{0}$ or after convergence, i.e., change in the $P_{i}$’s below a certain threshold:

(9) $$\begin{array}{cccc} k= & 1\cdots K_{0} & & \\ & i= & 1\cdots N & \\ & & S_{i}= & \left(E_{i}+\rho_{i}\sum_{j=1,j\neq i}^{N}P_{i}F_{ij}\right)/\left(1-\rho_{i}F_{ij}\right) \end{array}$$

(10) $$\begin{array}{cc} \;i= & 1\cdots N\\ & P_{i}=S_{i} \end{array}$$

Note that, for the similar Gauss–Seidel method, step 10 is omitted and $S_{i}$ is replaced by $B_{i}$ in step 9.

The Hapke reflectance model follows basically the same algorithm except that the isotropic intensity *I* in 3 needs to be replaced by the scattering phase function *P* of the form:

(11) $$\begin{array}{ccc} P(\mu ,\mu_{0},\Omega ) & = & \frac{\omega }{4(\mu_{0}+\mu )}\left[P\left(\Omega \right)\left(1+B\left(\Omega \right)\right)+H\left(\mu \right)H\left(\mu_{0}\right)-1\right] \end{array}$$

(12) $$\begin{array}{ccc} H(x) & = & \frac{1+2x}{1+2x\sqrt{1-\omega }} \end{array}$$

(13) $$\begin{array}{ccc} B(x) & = & \frac{B_{0}}{1+\tan(x/2)/h} \end{array}$$

Here, $\mu ,\mu_{0},\Omega$ denote the cosine of the inbound ray and the outbound ray and the phase angle between inbound and outbound ray, respectively. Additional surface parameters are represented by $\omega ,B_{0},h$, the single scattering albedo, and the height and angular width of the hot-spot correction.

#### Impact of Multiple Scattering

For the cases studied in this paper, the impact of multiple scattering is low. However, the runtime cost increases by a factor of 10–20 depending on solar azimuth and elevation. It should be noted that multiple scattering leads to an O(N${}^{2}$) complexity of our algorithm. Thus, the illumination model does not utilize this feature in the following analysis.

**Figure 7:**
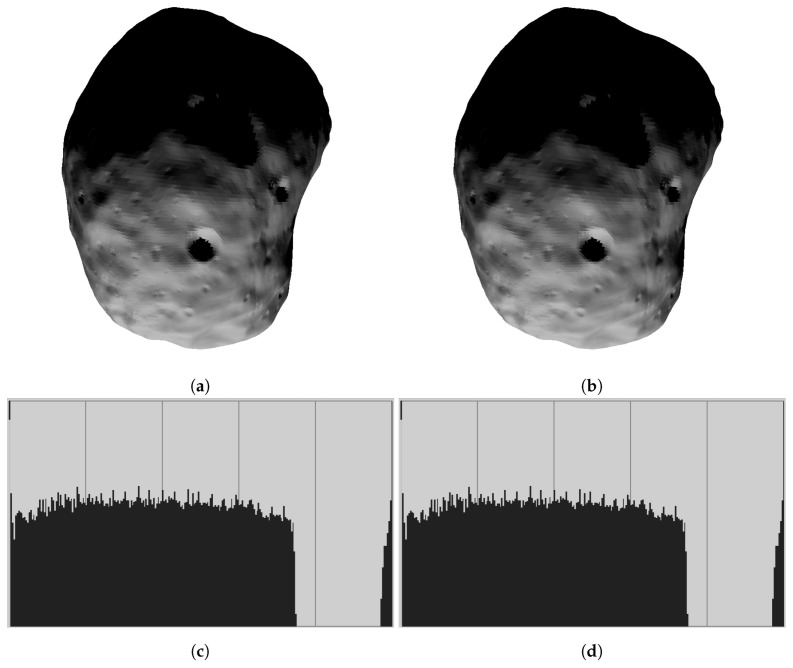
Comparison between irradiances with and without multiple scattering. (**a**) surface mesh, without multiple scattering; (**b**) Martian moon Phobos, 49152 triangle surface mesh, with multiple scattering. Maximal differences in irradiance compared to (**a**) are 2–3; (**c**) histogram corresponding to (**a**), multiple scattering toggled off; (**d**) histogram corresponding to (**b**), multiple scattering toggled on.

Figure 7a,b show illumination conditions (irradiances) on the Martian moon Phobo (approx. 49k surface triangles). Figure 7c,d show the corresponding image histograms. Note that effects of multiple scattering can be visible on the concave part on the surface only, and this applies in particular to crater slopes. A perfect sphere would show no difference for multiple scattering toggled on or off.

## 7. Remark on Multiple Bodies

**Figure 8:**
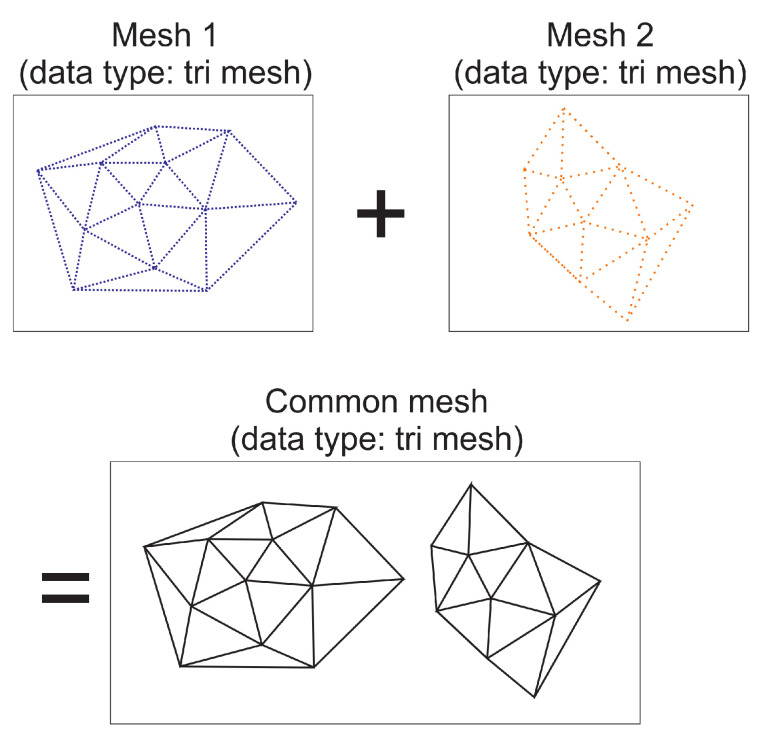
Multiple bodies’ triangle meshes are internally handled like a single mesh, thus allowing for easy implementation of mutual illumination or shadowing as well self-illumination (diffuse) e.g., in crater walls.

The algorithm does not distinguish between triangle patches of one or the other body. An arbitrary number of bodies can be handled simultaneously, i.e., illumination of one body by one (or more) other(s) can be handled by design. At the beginning of the algorithm, triangle meshes of the individual bodies are read and concatenated into a single triangle mesh (see Figure 8). For this mesh, generation of the AABB (see Section 6.1), direct illumination (Section 6.1) as well as diffuse illumination up to arbitrary orders of scattering (Section 6.2) is carried out. The same applies for mutual shadowing. This is in fact a part of the calculation of direct illumination.

## 8. Runtime Cost and Code Base

The runtime cost for generation of the Vesta images shown in this paper is approximately 17 s (single CPU i7-6700, 6th generation, no parallelization) and approximately 700 MB of RAM usage. This holds true for a shape model of 49k triangles (as shown in this paper) and a 10 × 10 × 10 spatial raytracing grid. Running times depend on O(N) and the number of triangles N. The size of the spatial raytracing grid mildly impacts the CPU time (see Table 3).

The more grid cells there are, the more often a ray crosses the border between two cells. This counteracts the beneficial effect of reduction of the number of triangles to be tested against intersection with the ray. The sweet spot seems to be somewhat around 10 × 10 × 10 cells. Other numbers such as 9 × 9 × 9 or 11 × 11 × 11 have not been tested, as the impact in in the single-digit second range.

The total code base (without comments) is only 12 k code lines, and is thus very small.

## 9. Exemplary Applications

### 9.1. Example: Phobos

**Figure 9:**
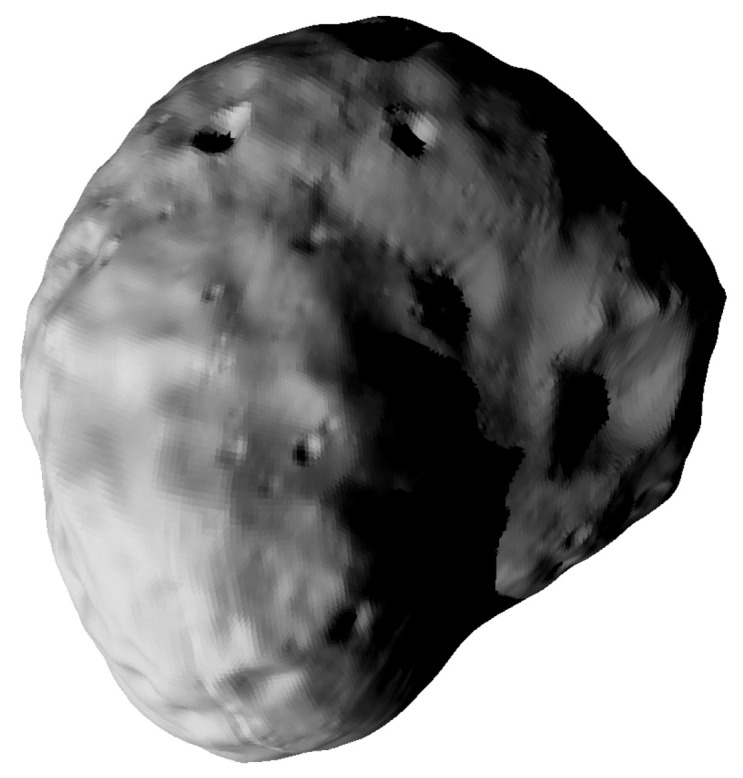
Solar illumination of Martian moon Phobos [17], showing realistic shadowing e.g., of craters.

**Figure 10:**
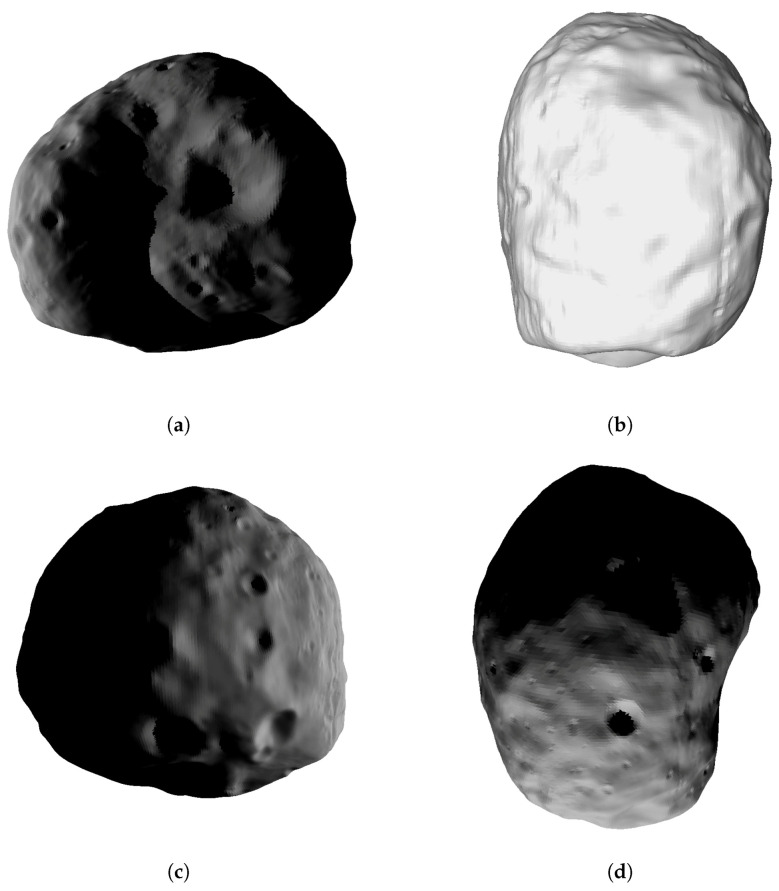
Solar illumination of Martian moon Phobos, showing realistic shadowing in different angles of view. (**a**) Phobos [17], solar radiation from the left-hand side; (**b**) Phobos [17], solar radiation from the front (directed into image plane); (**c**) Phobos [17], solar radiation from the right-hand side; (**d**) Phobos [17], solar radiation from the bottom.

The Martian moon Phobos has been illuminated, showing shadowing e.g., of crater floors, see Figure 9 and Figure 10. The spatial resolution of this mesh is 49,152 triangles, i.e., approximately 278 m average triangle edge length.

### 9.2. Mutual Shadowing

**Figure 11:**
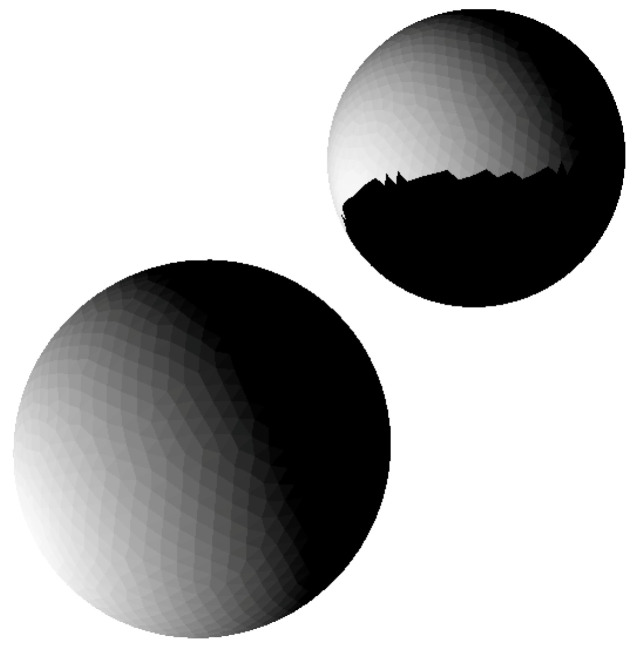
Shadowing of two spheres of radius 1200 km, each. Spheres are located at (0,0,0) and (−3000,1000,500), respectively. Direction to the sun is (1m,0,0). The blocky appearance of the shadow is a result of the discretization of the shadowed sphere.

Figure 11 shows mutual shadowing of two spheres. The two spheres are assumed to have equal radii of approx. 1200 km (comparable to Pluto, see below) and are equally meshed with 2048 and 4096 nodes and triangles each, respectively. One sphere is located at (0,0,0), whereas the other is located at (3000,1000,500), solar radiation is assumed to impinge from direction (1,0,0). The shadow of one sphere on the surface of the other is clearly visible, showing discretization artifacts at the border, however. Note that, in general, small bodies or lunar surfaces are available in much higher resolution, see Figure 2.

**Figure 12:**
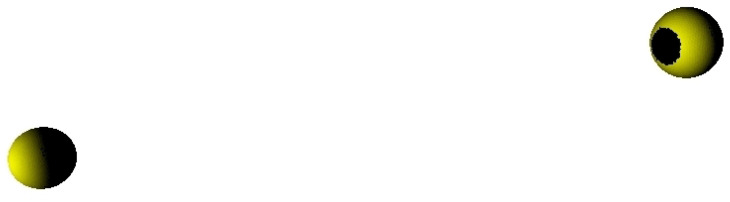
Shadowing of Pluto (right) by its largest moon Charon (left) during lunar eclipse (idealized). Both bodies are located on the *x*-axis, coordinates: Pluto (0,0,0), Charon (19,596,0,0), sun vector (1,0,0).

The Pluto–Charon binary system has been considered as a second example. Idealized positions of Pluto and Charon have been assumed, i.e., both bodies on the *x*-axis, separated by the real average distance of 19,596 km. Radii are 1187 km and 606 km for Pluto and Charon, respectively. Figure 12 shows the shadow casted by Charon on Pluto’s surface, assuming the sun in direction (1,0,0).

### 9.3. Illumination Statistic

The SLIM illumination model can be used to carry out statistical analysis, yielding the cumulative distribution function (cdf) for the irradiance at different triangle patches on the body surface. The cumulative distribution function assesses the times the irradiance is above some certain level. Usually, time intervals are calculated w.r.t to some total time, e.g., a full spin period:

(14) $$\begin{array}{ccc} F_{k}\left(x\right)\cdot T_{\mathrm{tot}} & = & \{t\in [0,\infty \left[\;:\;I_{k}\left(t\right)\leq x\right\} \end{array}$$

**Figure 13:**
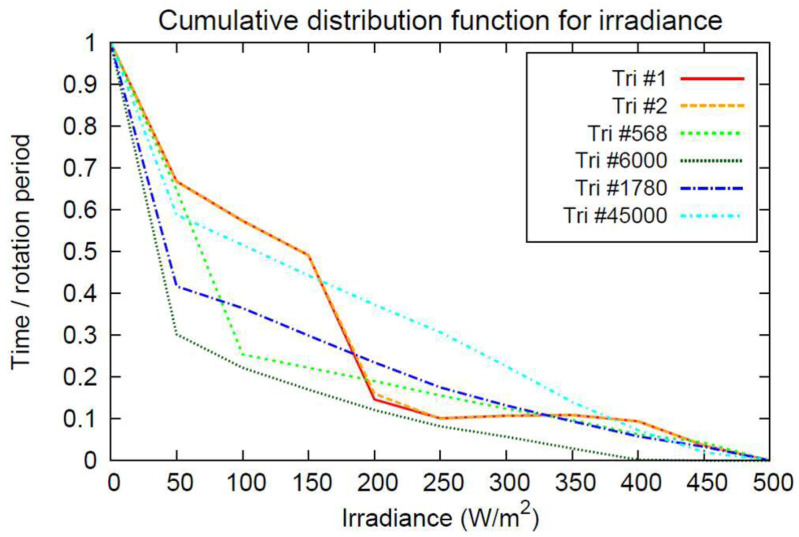
Cumulative distribution function for the irradiance at different points (triangle patches) on Phobos’ surface, relative to $T_{\mathrm{tot}}$ being Phobos’ spin period, i.e., 7.65 h. $F_{k}\left(x\right)$ is shown, see Equation (15) for five different triangles

In other words, the irradiance on triangle patch #*k* is smaller than some level *x* for ”$F_{k}\left(x\right)$%” of the total time. Figure 13 shows the results for Phobos, calculated for a full spin period of 7.65 h at 1.52 AU heliocentric distance and for zero obliquity. Exemplary cdfs are shown for five different triangles. Note that the maximal irradiance does not exceed approx. 500 W/m${}^{2}$.

## 10. Validation

A number of publications reported on optical properties of the surface of (4) Vesta as measured using the DAWN framing camera [15,24,25] (see Table 4). Also, landmark estimates from optical (as well as tracking) data have been investigated to derive dynamical properties such as gravity field, spin pole, and rotation period [26].

**Figure 14:**
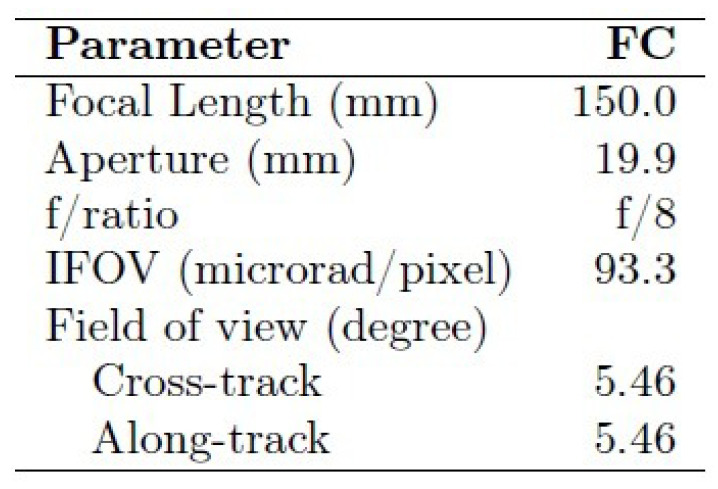
Basic parameters of Framing Camera 2 of the DAWN mission [24]. For the study presented here, data of the camera #ID 2 have been utilized.

**Figure 15:**
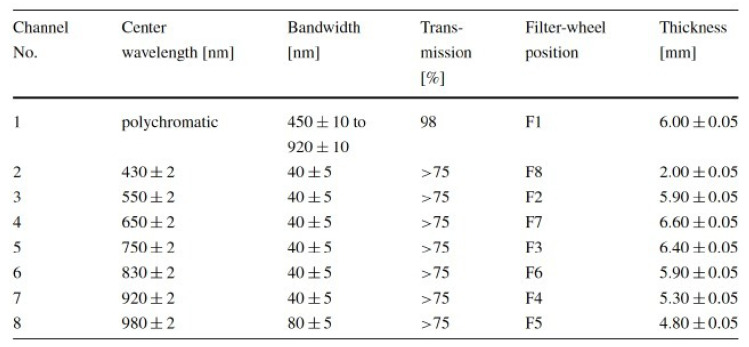
Filter settings of Framing Camera 2 of the DAWN mission [24]. For the study presented here, measurements using the clear filter have been utilized [24].

For validation purposes, a number of images acquired by the DAWN Framing Camera FC2 (see Figure 14 and Figure 15 for basic parameters) have been utilized. Corresponding ephemerides and pointing parameters have been provided by the SPICE toolkit in version N00066, released April 2017 [27].

**Figure 16:**
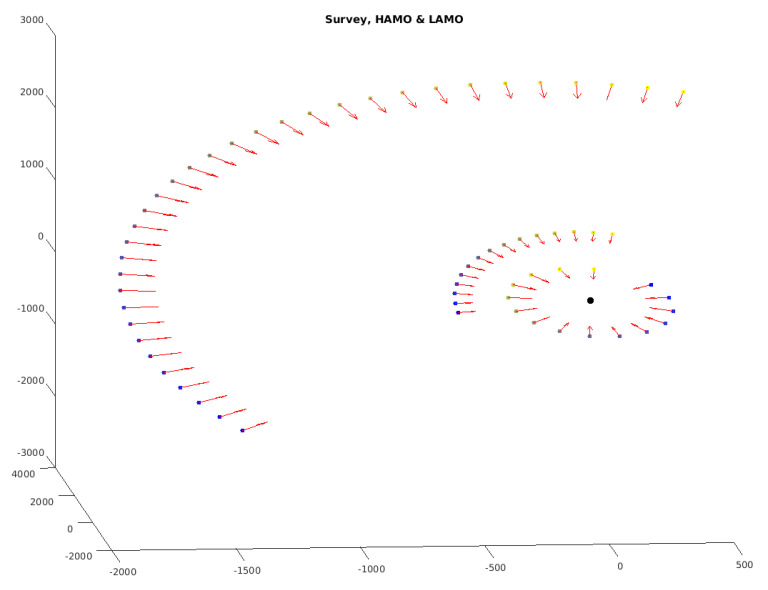
DAWN S/C positions relative to 4 Vesta during the survey, HAMO and LAMO phase. Orientations of the camera (FC 2) are shown as red arrows, and the color of each dot represents time during each mission phase from yellow (early) to blue (later) [18].

The validation data set (2665 pictures) is covering the time span of August 11–28 of the DAWN/Vesta survey data set, i.e., the mission phase covering overview pictures of 4 Vesta following the approach phase and followed by the high-altitude mapping orbit (HAMO) and low-altitude mapping orbit (LAMO) phase:

During the survey phase (see Figure 16), the average distance between the S/C and 4 Vesta is approx. 2720 km, and phase angles (angle between line-of-sight DAWN-Vesta and line connecting Vesta and the sun) cover the range from approx. 11 deg to approx. 81 deg.

The robustness of the proposed illumination model depends on the underlying shape model as well as on the BDRF reflectance function.

Firstly, uncertainties in the LTS, i.e., the direction of sunlight, are usually expressed as angular deviations. These uncertainties are independent from the underlying shape model or sizes of any features on the surface (craters, boulder, slopes).

The shape model introduces a spatial discretization error into the calculation. Given a certain absolute deviation (in meters, measured in local vertical direction, say), the relative error depends on the size of the feature. e.g., deeper craters suffer less from some absolute error than shallow craters.

**Figure 17:**
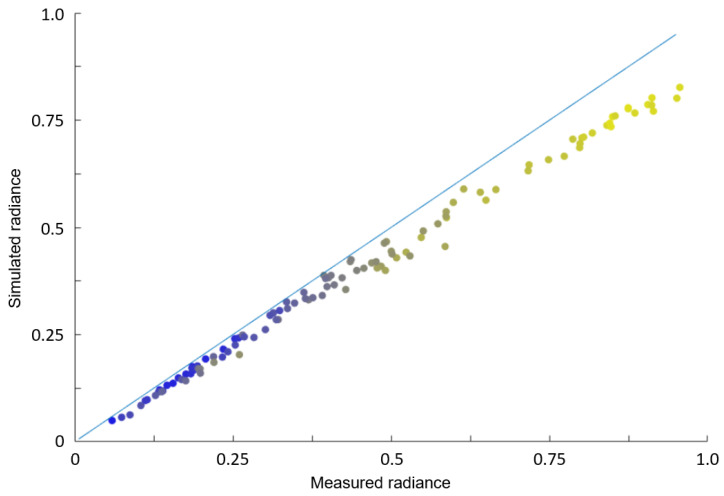
Comparison of real and measured radiance (scaled to [0,1], zero and one corresponding to minimal and maximal values, respectively), showing linear correlation, although with a slope below the theoretically expected slope of 1. Each data point represents the compared mean values of one picture. The colors indicate the solar zenith angle, and each dot represents a triangle (yellow=high, blue=low, ranging from 0 to 90 degrees), Image ID FC21B0007807-26232214089F1B.FIT. To improve readability, only deviations are detected for low zenith angles, thus indicating systematic deviations of the BDRF, especially for shallow illumination conditions. This might be a result of e.g., surface roughness not included in the Hapke BDRF used here.

**Figure 18:**
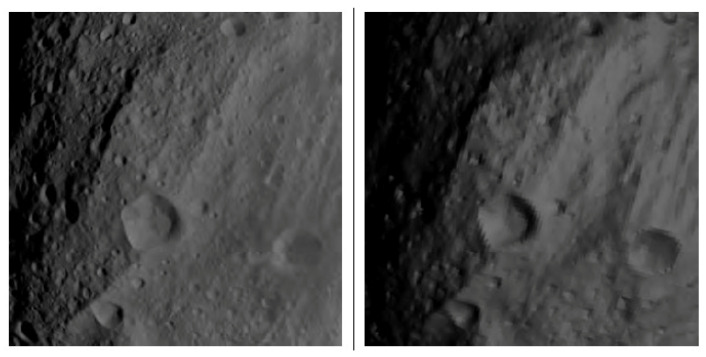
Left: real image as acquired by DAWN Framing Camera FC2. Right: simulated image, 196608-triangle-model of Vesta with 1024 × 1024 px camera resolution. Image ID FC21B0007807- 26232214089F1B.FIT, $\chi_{s}^{2}\approx 0.043$.

Figure 17 shows the correlation between simulated and measured radiance for image ID FC21B0007807-26232214089F1B.FIT (compare Figure 18. It can be observed that systematic deviation occurs for high solar zenith angles, indicating systematic deviations of the BDRF especially for shallow illumination conditions.

It shows that the accuracy of light and shadow in craters is very high; however, the contrast in the simulated picture seems to be stronger than in the real picture. This might be a result of the left-out surface roughness in the Hapke model. In addition, the edge of Vesta against the sky is a lot brighter in the real picture than in the model. To test the accuracy of the simulation, the proportion of irradiance arriving at Vesta to radiance received at the camera was calculated with the real data and the simulated data (Figure 17). An expected linear correlation can be found. However, for shallow illumination conditions (high solar zenith angles), the deviation increases.

**Figure 19:**
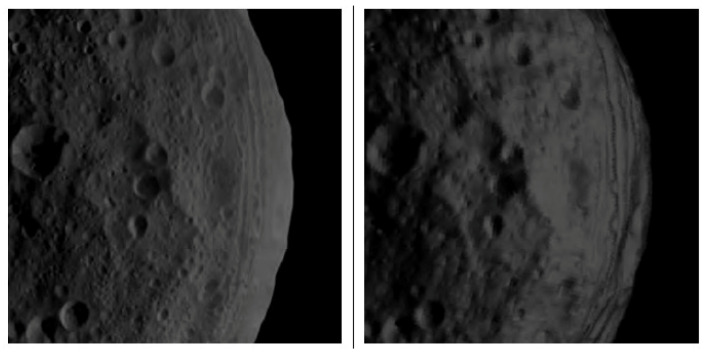
Left: real image as acquired by the DAWN Framing Camera FC2. Right: simulated image, 196608-triangle-model of Vesta with 1024 × 1024 px camera resolution. Image ID FC21B0005907- 11232212051F1B.FIT, $\chi_{s}^{2}\approx 0.039$.

Figure 19 shows another example of a successfully passed $\chi^{2}$ test (0.39).

**Figure 20:**
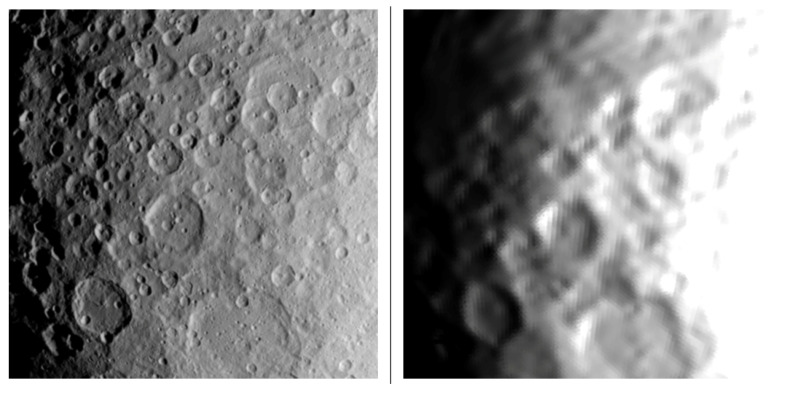
Left: real image as acquired by DAWN Framing Camera FC2. Right: simulated image, 196608-triangle-model of Vesta with 1024 × 1024 px camera resolution. Image ID FC21B0008907- 24563695343F1B.FIT, $\chi_{s}^{2}\approx 0.074$.

Figure 20 shows an example of a pair of simulated and measured image not passing the $\chi^{2}$ test (0.074). Obviously, the images do not match very well, and substantial differences in local illumination are visible.

### Statistical Analysis

**Figure 21:**
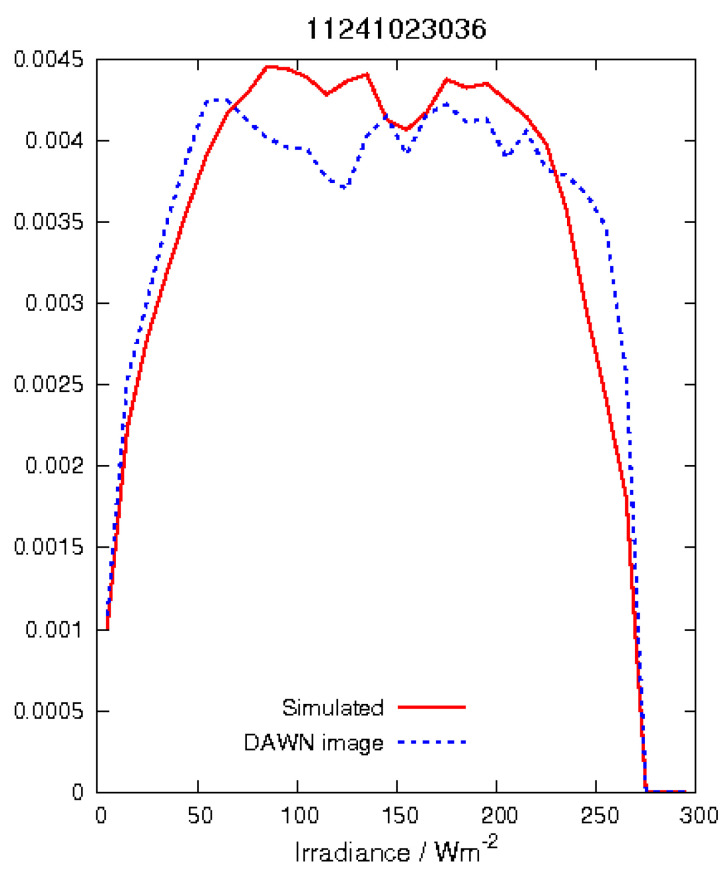
Exemplary radiance probability density function of image ID 11241023036. The DAWN image was acquired on 31 August. The RPDF shows an average radiance of approx. 151 W/sr/m${}^{2}$ and minimal and maximal values of 0. (black pixel or deep shadow) and 260 W/sr/m${}^{2}$, respectively. The radiance bins are 10 W/sr/m${}^{2}$.

Simulated and acquired images are compared by utilizing the radiance probability density distribution (RPDF). Measured respectively simulated radiances have been binned into 10 W/sr/m${}^{2}$ bins. An exemplary RPDF is shown in Figure 21.

The validation approach followed in this study is based on the comparison of simulated and acquired (measured) RPDF. This is done using a $\chi^{2}$ test. The test method is briefly outlined as follows. Assume a given RPDF with absolute frequencies $f_{1},\cdots ,f_{n}$ for the various radiance bins. This is represented by the RPDF of the measured image. Assume that $p_{1},\cdots ,p_{n}$ is the relative frequencies of the RPDF to be compared with. Given *N* samples pixels in this case), the deviation between the two RPDF can be quantified by the $\chi_{s}^{2}$ value:

(15) $$\chi_{s}^{2}=\sum_{k=1}^{N}\frac{{\left(f_{k}-Np_{k}\right)}^{2}}{Np_{k}}$$

The $\chi_{s}^{2}$ test statistic is an overall measure of how close the observed frequencies $f_{k}$ are to the expected frequencies $p_{k}$. Obviously, the ’null hypothesis’ (PDF and independent, i.e., simulated and measured image are not similar at all) is rejected if $\chi^{2}$ is large because this means that observed frequencies and expected frequencies are far apart. A quantification of ’large’ is acquired by the $\chi^{2}$ probability density function:

(16) $$f\left(x\right)=\frac{x^{N/2-1}e^{-x/2}}{2^{N/2}\Gamma \left(N/2\right)}$$

Here, *N* represents the degrees of freedom, identical with the number of pixels in our case. The $\chi^{2}$ curve is used to judge whether the calculated test statistic is large enough. We reject the null hypothesis if the test statistic is large enough so that the area beyond is less than 0.05:

(17) $$P\left(\chi^{2}\geq \chi_{s}^{2}\right)=\int_{\chi_{s}^{2}}^{\infty }f\left(\chi^{2}\right)d\left(\chi^{2}\right)$$

**Figure 22:**
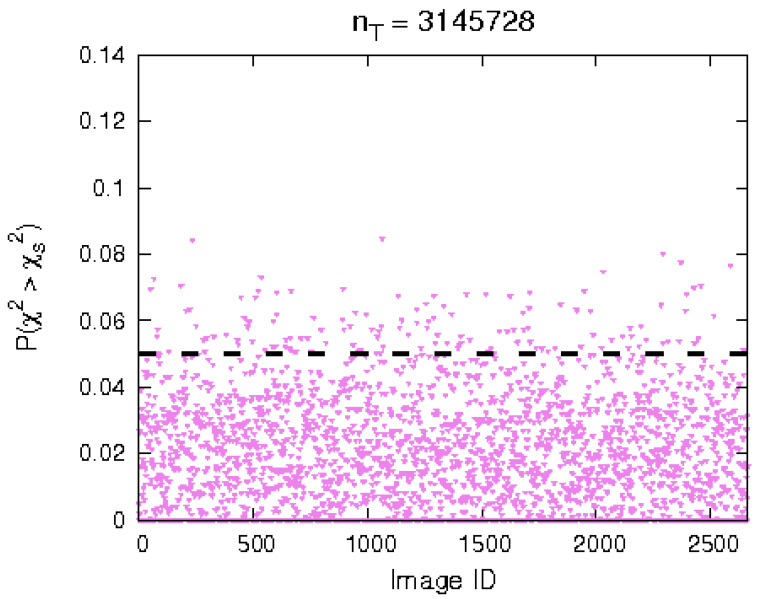
Results of $\chi^{2}$ test, 3,145,728 surface triangles.

This test is done for every picture and for every resolution available for the Vesta surface grid (see Table 1). As can be seen in Figure 22, the majority of the test cases yield values of $P(\chi^{2}\geq \chi_{s}^{2})<0.05$, i.e., the null hypothesis is rejected. Note that the null hypothesis is actually that the underlying RPDF of the acquired and simulated images are different. Thus, the reliability of the algorithm is good.

## 11. Parameter Estimation

The SLIM tool has been designed to be lightweight and efficient to be utilized in inverse modeling and parameter estimation for planetary surfaces without atmospheres. This applies to the lunar surface as well as to small body surfaces. The following sections presents a brief outlook into this use case (see Table 5).

**Figure 23:**
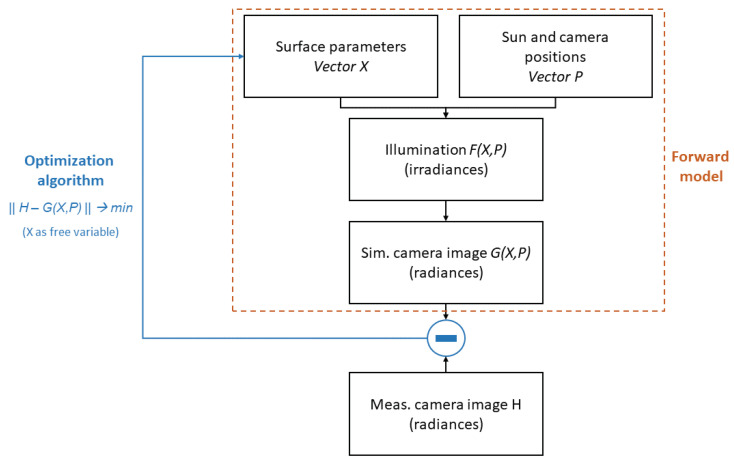
Algorithmic scheme for parameter estimation. The inversion should be done with a large amount of images to increase. The overall method is modular, that is, e.g., the optimization algorithm can be replaced by another one. In addition, the tool for generation of illumination conditions as well as that for camera images can be replaced by another one, as long as all interfaces are maintained.

In this study, the Hapke BDRF as given in Equation (11) is utilized, parameters to be estimated are B,ω,h. A sophisticated optimization algorithm has not been applied yet, but rather a simple brute-force approach has been taken. That is, every of the three parameters has been varied by using an interval bisection algorithm with approximately 1000 initial samples in the range [0,1] (ω and *h*) and [0,2] for *B*, respectively. Figure 23 shows the principal algorithmic scheme.

## 12. Conclusions and Outlook

An efficient raytracing software for simulation of camera images as well as illumination conditions of small bodies as well as planetary surfaces has been implemented. Direct as well as indirect illumination as well as illumination of multiple bodies are possible. Different bidirectional reflectance functions are implemented. This includes the four-parameter Hapke model.

The illumination model has been validated using a $\chi^{2}$ statistical test to compare simulated images with a large number of DAWN acquired images. A good statistical match is found in general.

The main purpose of SLIM is not the generation of realistic synthetic images, and the resolutions presented in the synthetic images in this paper are fairly low. The main benefits, however, of the simulation suite are its easy implementation and the option to use it as a forward model in an inverse problem solver to derive optical properties of the surface of small bodies or planetary surfaces (without atmosphere).

Using this illumination model, optical parameters can be extracted from the comparison of simulated and observed radiances and applications of an optimization algorithm to those data. Images in different spectral bands can be easily computed by restriction of the solar spectrum to the spectral region under consideration. This way, dependence on the spectral parameters on wavelength can be examined.

This has non-zero intersection with inverse problem theory, as the measured images might be noisy and the simulated images are restricted due to finite discretization of the surface, either a small body or any other planetary surface.

## Figures and Tables

**Table 1:** Available resolutions of surface grid of 4 Vesta [15].

Resolution	No. of Triangles	Average Edge Length
coarse	49,152	6744.7 m
medium	196,608	419.2 m
fine	786,432	28.3 m
super-fine	3,145,728	1.7 m

**Table 2:** SPICE kernels used in this study.

DAWN trajectory	dawn-rec-160617-160902-161109-v1.bsp
DAWN S/C orientation	dawn-sc-160104-160110.bc, dawn-sc60613-160619.bc
(4) Vesta trajectory	sb-vesta71107.bsp
(1) Ceres trajectory	sb-ceres-140724.bsp

**Table 3:** Runtime cost for different sizes of the raytracing grid.

Size Grid	CPU Time (49k Triangles)/s
1 × 1 × 1	32
2 × 2 × 2	29
5 × 5 × 5	23
10 × 10 × 10	17
20 × 20 × 20	21
100 × 100 × 100	35

**Table 4:** DAWN mission phases in Vesta’s vicinity [28].

Time	Mission Phase
Jul 16, 2011	Vesta arrival
Aug 11–31, 2011	Vesta survey phase
Sep 29, 2011–Nov 2, 2011	Vesta first high altitude orbit (HAMO)
Dec 12, 2011–May 1, 2012	Vesta low altitude orbit (LAMO)
Jun 15, 2012–Jul 25, 2012	Vesta second high altitude orbit (HAMO)
Sep 5, 2012	Vesta departure

**Table 5:** Parameter estimation study using a brute force approach for Hapke parameters B,ω,h. Values of *B* and ω show fairly good agreement, whereas parameter *h* is not in good agreement with the literature values [15,25].

Parameter	This Study	[25]	[15]
	Image ID FC21B0005907-11232212051F1B.FIT	(4) Vesta Average	(4) Vesta Average
*B*	1.83	1.7	1.0
ω	0.45	0.51	0.51
*h*	0.53	0.07	0.098
